# Solitary Bone Plasmacytoma Mimicking Oral Cavity Neoplasm – A Rare Case

**DOI:** 10.1007/s12070-023-03875-2

**Published:** 2023-05-17

**Authors:** Vikrant Kamboj, Vijendra Shenoy S, Susmita Sriperumbudur, Navya Parvathareddy

**Affiliations:** Department of Otorhinolaryngology and Head and neck Surgery, Kasturba Medical College, Mangalore Manipal Academy of Higher Education, Manipal, India

**Keywords:** Plasma cell tumour, Plasmacytoma, Oral cavity neoplasm

## Abstract

Plasmacytomas are localized monoclonal plasma cell lesions with no evidence of systemic involvement which are divided into solitary bone plasmacytoma (SBP) and extra-medullary plasmacytoma (EMP). The diagnosis of plasmacytomas (PCM) in the oral regions is challenging given the atypical clinical manifestations and low frequency. Here, we report an extremely rare case of plasmacytoma in an elderly male which initially appeared to be arising from the left buccal mucosa on clinical examination but after radiological imaging and intra-operative findings, the epicentre was found to be in the left infratemporal fossa (ITF). The patient underwent en-bloc compartment resection with high clearance of the ITF which proved to be an effective management strategy. It is crucial for the head and neck surgeon to be aware of the solitary bone plasmacytoma in the oral and maxillofacial region in order to identify it early and provide these patients with the best care possible before complications arise.

## Introduction

Plasma cell tumours are classified into multiple myeloma and plasmacytomas. Plasmacytomas are localized monoclonal plasma cell lesions with no evidence of systemic involvement which are divided into solitary bone plasmacytoma (SBP) and extra-medullary plasmacytoma (EMP) [1]. Most common sites for SBP are long bones, vertebrae, sternum, clavicle, rib and humerus [2]. It rarely involves maxillofacial region with mandible (especially ramus, body, angle due to marrow-rich areas) involved in 4.4% cases of SBP. EMP, also known as extra-osseus solitary plasmacytomas develop outside the bone marrow on mucosa of upper respiratory and digestive tracts such as nasal sinuses, oropharynx and larynx [1].

The diagnosis of plasmacytomas (PCM) in the oral regions is challenging given the atypical clinical manifestations and low frequency. Therefore, identification of the clinical, radiographic, and histopathological features of this neoplasm is of immense importance to head and neck surgeons to make an appropriate diagnosis. Here, we report an extremely rare case of plasmacytoma which initially appeared to be arising from the left buccal mucosa on clinical examination but after radiological imaging and intra-operative findings, the epicentre was found to be in the left infratemporal region.

## Case Report

A 65-year-old male presented to our hospital with complaint of swelling over the left cheek with swelling under the left eye since the past 6 months. The swelling was associated with pain since the last one month which was intermittent in nature and pricking in character which aggravated with jaw movements and relieved with oral painkiller. On examination of the oral cavity, a 4*3 cm submucosal growth was palpable in the left buccal mucosa extending anteriorly 2 cm short of oral commissure, posteriorly involving the retromolar trigone with upper and lower gingivo-buccal sulcus free of the lesion. The lesion had a smooth surface, well-defined margins, non-tender, non-mobile and there was no evidence of cervical lymphadenopathy.

**Fig. 1:**
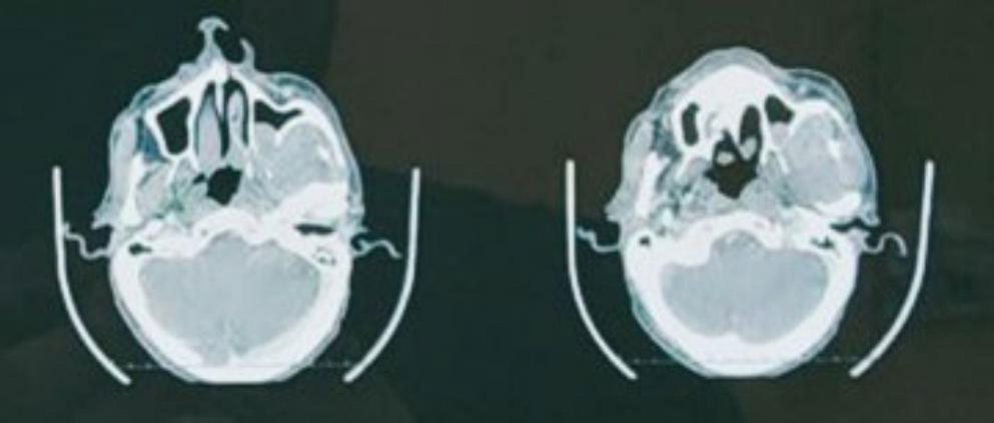
CT imaging in axial section showing the extension of lesion into left infratemporal region with erosion of pterygoid plates

**Fig. 2:**
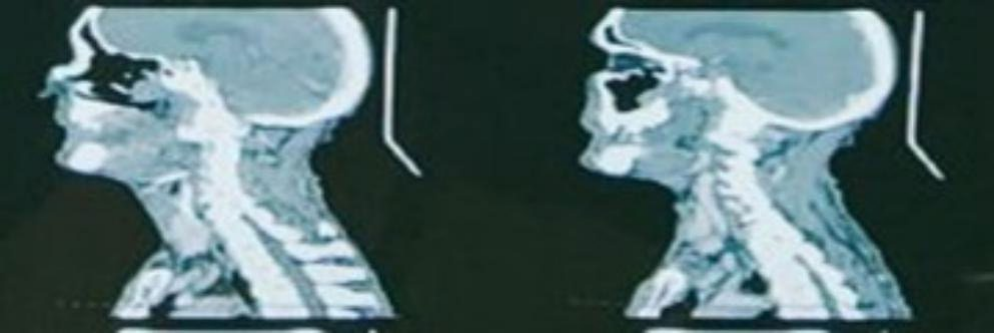
CT imaging showing extension of lesion into left pterygopalatine fossa with extension into left maxillary sinus eroding its postero-lateral wall

Biopsy of the growth was done on out-patient basis which was reported as normal study. Later, tru-cut biopsy of the lesion reported it as a salivary gland tumour. Contrast enhanced Computed Tomography (CT) scan showed an evidence of lobulated infiltrative soft tissue attenuation mass, possibly malignancy, approximately 3.7*3.4*5.5 cm epicentered in the left infratemporal region invading into the left masticator space, pterygopalatine fossa, with associated erosion of pterygoid plates. (Fig. 1) Medially it extended into the posterior nasal cavity and anteriorly into left maxillary sinus with complete erosion of its postero-lateral wall, and superiorly up to inferior orbital fissure without intra-orbital extension. (Fig. 2) There was no involvement of the mandible and no intracranial extension. Since, the mouth opening was adequate i.e., 3 finger breadth on clinical examination, it was assumed that pterygoid muscles were not involved.

**Fig. 3:**
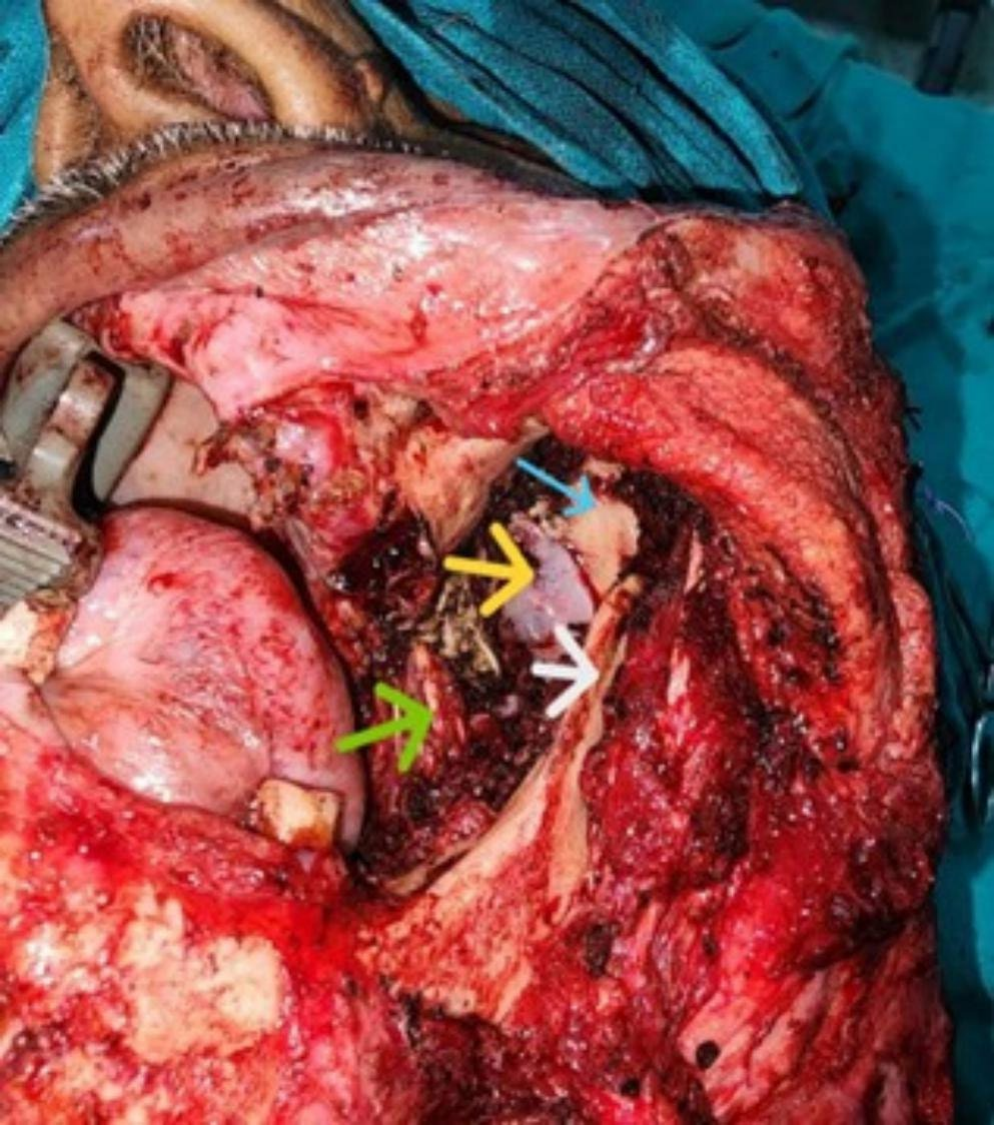
Intra-operative photograph of the left infratemporal fossa showing the anatomical landmarks after compartment resection. (Blue arrow: Greater wing of sphenoid, yellow arrow: foramen ovale, green arrow: medial pterygoid, white arrow: coronoid process)

**Fig. 4:**
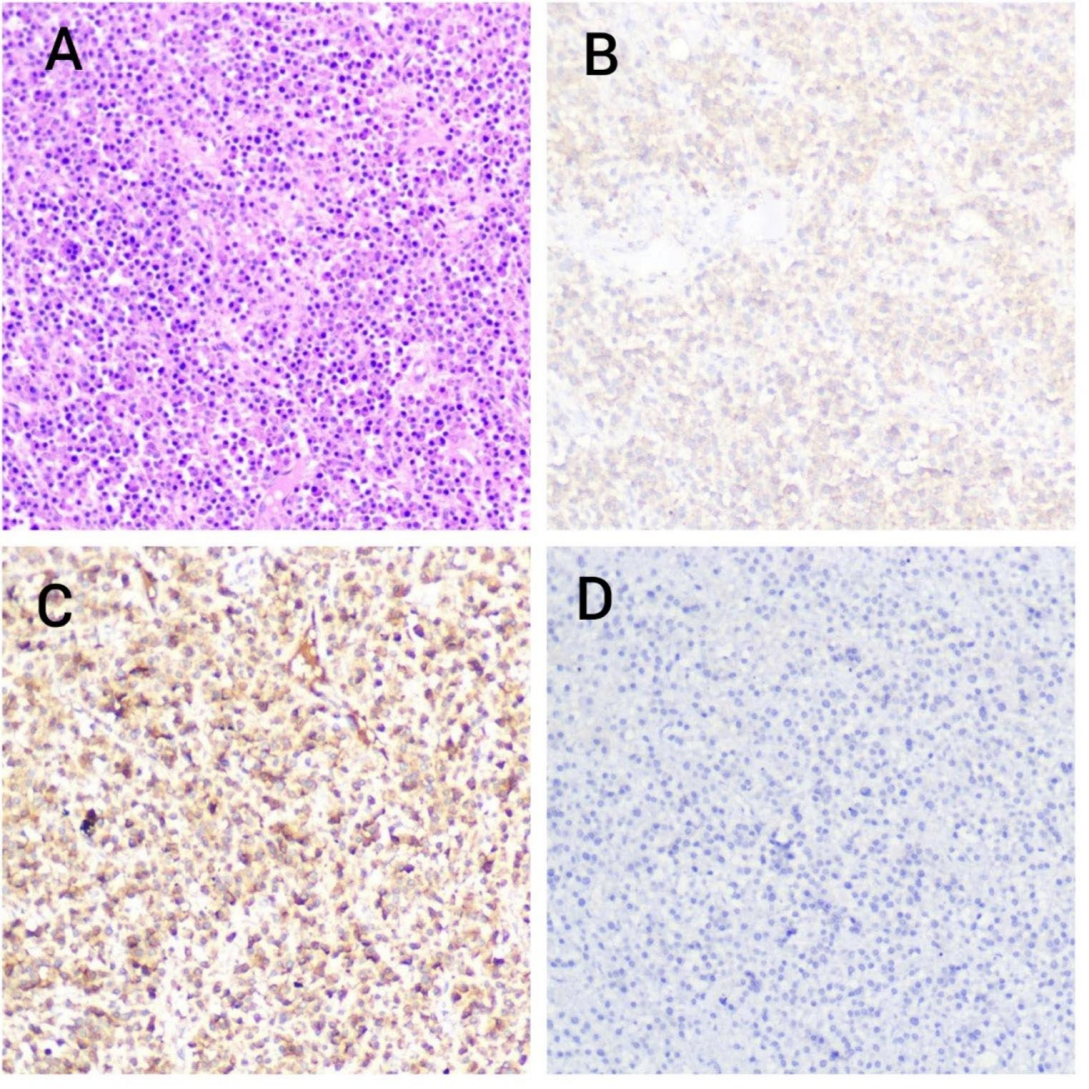
Photomicrographs showing (A) large, round/oval shaped plasma cells with vesicular nuclei, H&E 40x (B) CD 138 positive (C) Anti-kappa light chain positive (D) Anti-kappa light chain negative

The patient underwent En-block compartment resection with high clearance of ITF. In this case, ITF clearance was done while preserving the mandibular condyles as they were not involved. (Fig. 3) The specimen was sent for histopathological examination which revealed large round/oval malignant plasma cells with vesicular nucleoli. The Immunohistochemistry markers were positive for CD138 and Kappa, thereby confirming the diagnosis of Plasmacytoma. (Fig. 4)

## Discussion

Plasmacytoma was first described by Schridde, in 1905, as an uncommon solitary growth of neoplastic monoclonal plasma cells [3]. The International Myeloma Working Group lists three types: solitary plasmacytoma of bone (SPB), extramedullary plasmacytoma (EMP) and multiple plasmacytomas, with SPB being the most common of these, accounting for 3–5% of all plasma cell malignancies [4]. The incidence rate of plasma cell tumour is about 2.6 to 3.3/lac population in the world with slightly higher preponderance in African-Americans [1]. They usually affect elderly age group of 50–80 years with a male: female ratio of 3:1.

The aetiology of oral cavity plasmacytoma is unknown, but risk factors include exposure to chemicals, radiation, viral infection (Epstein-Barr virus in immunocompromised patients) chronic inflammation, and genetic disorders [5], [6].

Clinical presentation of plasmacytomas may be asymptomatic or non-specific like pain and swelling near the affected bone (SPB). EMPs most frequently arise from upper respiratory tract mucosa, causing symptoms such as epistaxis, rhinorrhoea, and nasal obstruction but they can arise in any soft tissue, including oral cavity mucosa [7]. It is important to differentiate them from other soft tissue tumours and neoplasms of the oral cavity.

Systemic plasma cell disorders such as multiple myeloma have increased blood calcium, deranged renal function, anaemia and multiple bone lesions (CRAB syndrome) which is not seen in true plasmacytoma [8]. However, there is a chance of SPBs progressing to multiple myeloma over a period of 4–5 years. Since the management for both varies with radiotherapy being the mainstay for plasmacytomas (surgery in specific cases), and chemotherapy, stem cell transplant for multiple myeloma, it is necessary to distinguish between them.

The present case report describes a 65-year-old male patient with a suspected salivary gland tumour arising from the left infratemporal fossa (ITF). Based on CECT and tru-cut biopsy, the patient was planned for Compartment resection with ITF clearance. Since mouth opening was adequate on examination, mandible not involved on imaging, and appeared free of tumour intra-operatively, the mandible condyles were spared. As routine blood investigations including renal function tests were normal. Serum protein electrophoresis and urine analysis for Bence Jones protein was done post-operatively and showed normal study.

On histology, these tumours show sheets of plasma cells with varying differentiation of atypical plasma cells (i.e. abundant cytoplasm with eccentric located nuclei), inclusion bodies and cartwheel chromatin pattern [2]. CD138 is a marker for plasma cells, plasma blasts, and some immunoblasts. Immunohistochemistry demonstrates both kappa or lambda immunoglobulin light chain in inflammation because plasma cells are polyclonal. But SBP has either kappa or lambda light chain, since atypical plasma cells are monoclonal in nature. The present case showed CD 138 positivity with kappa positivity thereby confirming the diagnosis of SBP.

The ITF is a complex anatomical region containing vital structures such as the maxillary artery, pterygoid muscles, and trigeminal nerve branches. Tumours in this region are rare and account for less than 1% of all head and neck malignancies. The surgical approach to ITF tumours is challenging due to the complex anatomy and proximity to vital structures. ITF tumours require a multidisciplinary approach involving otolaryngologists, maxillofacial surgeons, and neurosurgeons. Different surgical approaches include open or endoscopic, transcervical, transmandibular, or transmaxillary routes and high ITF approaches [9]. The choice of surgical approach depends on the extent of tumour invasion and the involvement of adjacent structures [10]. In the present case, high ITF clearance with en bloc compartment resection was performed due to the extent of tumour invasion.

Imaging plays a crucial role in diagnosing and managing Contrast-enhanced computed tomography (CECT), and magnetic resonance imaging (MRI) is the chosen imaging modality. CECT is preferred for initial evaluation due to its wider availability, better resolution of bony structures, and ability to detect calcifications. MRI is preferred for evaluating soft tissue and neural involvement and planning the surgical approach [11].

Metastatic lesions, osteoblastomas, Ewing sarcomas, rhabdomyosarcomas, and histiocytomas are a few differential diagnoses for SBP. Osteolytic osteoblastic metastatic lesions have localised radiolucency that resembles SBP.

Plasmacytoma is a radiosensitive tumour and hence, radiotherapy is the main choice of treatment for both SPB and extramedullary plasmacytoma. Used with curative intent, and local control rates of > 80% can be achieved [12, 13]. Fractionated radiotherapy dose of 40–50 Gy over a duration of 4 weeks is given at the rate of 1.8 to 2.0 Gy per fraction [12]. Chao et al. found that surgical resection, either alone or in combination with adjuvant irradiation, proved a better pattern of response to EMPs originating in the head and neck [14]. The role of angiogenesis inhibitors, thalidomide, protease inhibitors, or inhibitors of vascular endothelium growth factor in plasma cell neoplasms is now the subject of further research.

Nearly 80% of SBP patients do not experience any recurrences even after five years, while 50–80% of individuals survive SBP for ten years [1]. The likelihood that SBP will develop into MM depends on a few risk variables, including age (> 60 years), M component levels > 20 g/L up to one year after radiation, tumour size (> 5 cm), and vascularity [3]. Regular following up of SPB cases is therefore mandatory after treatment.

## Conclusion

Solitary bone plasmacytoma is a lymphoproliferative disorder that involves clonal proliferation of plasma cells. Our case reports an extremely rare case of plasmacytoma presenting as a submucosal swelling in the oral cavity but epicentered in the left ITF. Compartment resection with high ITF clearance is an effective surgical approach for such lesions.

Although the clinical and radiographic signs of SBP might not be non-specific, clinicians should always keep them in mind when making a differential diagnosis for oral cavity lesions. It is crucial for the head and neck surgeon to be aware of the solitary bone plasmacytoma in the oral and maxillofacial region in order to identify it early and provide these patients with the best care possible before complications arise.
